# Annotations

**Published:** 1903-11-28

**Authors:** 


### ANNOTATIONS.

### Births, Marriages, and Deaths.

THE quarterly return of the Registrar-General is again with us, and again it tells a tale of diminished mortality which is eminently satisfactory, but which, in view of the character of the season through which we have been passing, is not at first quite easy to understand. There is only too much evidence that the excessive rainfall has been injurious to vegetable life and to many animals; but the human race, at least in these islands, appears to have thriven upon it in a very remarkable manner. The deaths registered in England and Wales during the last quarter were only in the proportion of 13·9 annually per thousand persons living, against an average rate for the ten preceding third quarters of 16·9. The mortality of males showed a decrease of 3·2, and that of females a decrease of 2·9, per thousand living, compared with the average as above. The mortality of infants under one year was 133 per thousand, as against an average of 194; while the death-rate of persons between one year and 60 showed a decrease of 14·8 per cent., and that of persons over 60 a decrease of 1·5 per cent. The mortality from the seven epidemic diseases showed a definite reduction in all but small-pox, which occasioned a number of deaths slightly in excess of the corrected average. The birth-rate of the quarter has again somewhat declined, from 29·3 per thousand of the population to 28·7; but we are happy to add that the marriage-rate has increased from 16·8 to 17·5, so that there is at least an increasing prospect of the birth-rate being restored.

### The Notification of Infectious Diseases.

THE public prints continue every now and again to record illustrations of the inherent difficulties of the notification of infectious diseases under the existing regulations. Only the other day a practitioner was summoned for failing to notify a case of small-pox which he had diagnosed as chicken-pox, and in support of the prosecution it was argued that if doubt existed it was the duty of the practitioner to communicate with the medical officer of health. The magistrate rightly rejected this contention, and indeed the form of the prescribed certificate is sufficient in itself to show that, not suspicion, but a definite opinion is the only justification for the notification of any individual case. The health authorities in more than one instance have refused—and quite rightly so far as the Act of Parliament is concerned—to take any share in the steps necessary for a diagnosis. The entire responsibility for these is thrown on the shoulders of the medical attendant and it is only when he is ready to sustain the onus of a definite opinion that the case comes under the official notice of the sanitary authority. In these circumstances the first claim on the practitioner is the protection of the patient and his own security. He is not a public official or sanitary detective but a private medical adviser. Now it is certainly not in the interest of the patient to be labelled as the subject of an infectious disease until the facts leave no moral doubt that this is a correct diagnosis. Equally it is an act full of risk to pronounce on inadequate evidence a diagnosis which may involve serious prejudice to the patient and his affairs. If the public interest demands the early notification of doubtful or suspicious cases, the responsibility for this must be borne not by the private practitioner but by public authority. It would no doubt tend to prevent the spread of infectious diseases were cases notified on the basis of "reasonable suspicion." The existing Act does not provide for this and indeed opposes early notification, because under it the practitioner cannot safely act until the evidence is conclusive. If in his anxiety for the public health he notifies a case before conviction is complete and subsequent events prove him to have been mistaken the law leaves him to bear all the consequences. What ought to rest on the sanction of official responsibility is in present circumstances placed on the shoulders of the individual practitioner. He is encouraged to undertake public duty and to perform public service with the risk that at the critical moment he will be left without public support.

### Facial Paralysis following Emotion.

THE etiological problem presented by facial paralysis in the ordinary form of Bell's palsy is one which is far from being provided with a satisfactory solution. Of recent years the "chill" theory, which formerly was so confidently applied to many clinical events, has suffered a considerable measure of eclipse, and the term "rheumatic" has in almost equal degree been placed in the category of words without knowledge. This at least is the position adopted by the exponents of advanced pathological and bacteriological doctrine. It may be that the older views, based upon wide experience rather than on scientific demonstration, have a stronger claim than their modern contemners recognise, but even those who hold them will allow that they cannot be advanced as absolute truths. There is, therefore, abundant justification for the record of all cases in which the sequence of events suggests a causal relation between such an occurrence as a facial paralysis and some preliminary phenomenon. It is this consideration which renders particularly interesting the description by Dr. Leonard Williams of a case of Bell's palsy, in which all suggestion of a preceding chill and of personal rheumatic tendencies can be excluded. The interest is further increased by the fact that the paralysis immediately followed a fright. The patient was a woman of 27 years. After being greatly alarmed one evening by her baby falling downstairs, she was found on the following morning to be the subject of a left-sided facial paralysis. Dr. Williams suggests that an analogy to this experience may be found in cases of chorea, in which it may be presumed that emotion acts as an exciting cause and rheumatism as a predisposing factor. The absence of a history of rheumatism from his case to some extent weakens this suggestion, but, of course, does not negative it in view of the subtle character now widely credited to the rheumatic poison. There is the further resemblance between Dr Williams's case and chorea that in not a few instances of the latter disease the weakness of the affected muscles is much more pronounced than the clonic spasm. Indeed, every now and then this weakness is so extreme that the case is liable to be mistaken for hemiplegia or other form of paralysis. In any view of the matter it must in future be recognised that mental emotion requires consideration among the possible causes of facial paralysis.

